# Supplementary material for: Causal Relationship between Aging and Anorexia Nervosa: A White-Matter-Microstructure-Mediated Mendelian Randomization Analysis
Source: Biomedicines. 2024 Aug 16;12(8):1874. doi: 10.3390/biomedicines12081874 (PMC11351342; doi:10.3390/biomedicines12081874)
Supplement: Supplementary file 1 [file biomedicines-12-01874-s001.zip › Supplementary Figure/Figure S4/Singe SNP plot of MR analysis for TL to ubm-b-2021.pdf]

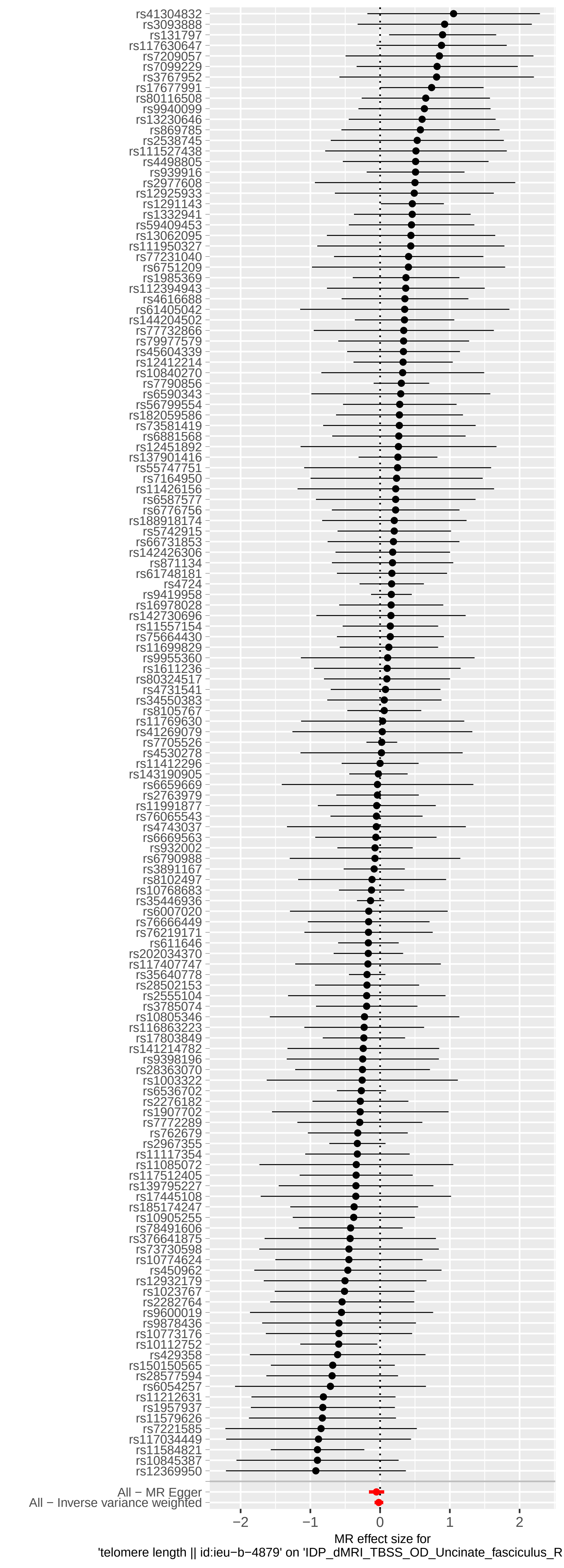

rs41304832
rs3093888
rs131797
rs117630647
rs7209057
rs7099229
rs3767952
rs17677991
rs80116508
rs9940099
rs13230646
rs869785
rs2538745
rs111527438
rs4498805
rs939916
rs2977608
rs12925933
rs1291143
rs1332941
rs59409453
rs13062095
rs111950327
rs77231040
rs6751209
rs1985369
rs112394943
rs4616688
rs61405042
rs144204502
rs77732866
rs79977579
rs45604339
rs12412214
rs10840270
rs7790856
rs6590343
rs56799554
rs182059586
rs73581419
rs6881568
rs12451892
rs137901416
rs55747751
rs7164950
rs11426156
rs6587577
rs6776756
rs188918174
rs5742915
rs66731853
rs142426306
rs871134
rs61748181
rs4724
rs9419958
rs16978028
rs142730696
rs11557154
rs75664430
rs11699829
rs9955360
rs1611236
rs80324517
rs4731541
rs34550383
rs8105767
rs11769630
rs41269079
rs7705526
rs4530278
rs11412296
rs143190905
rs6659669
rs2763979
rs11991877
rs76065543
rs4743037
rs6669563
rs932002
rs6790988
rs3891167
rs8102497
rs10768683
rs35446936
rs6007020
rs76666449
rs76219171
rs611646
rs202034370
rs117407747
rs35640778
rs28502153
rs2555104
rs3785074
rs10805346
rs116863223
rs17803849
rs141214782
rs9398196
rs28363070
rs1003322
rs6536702
rs2276182
rs1907702
rs7772289
rs762679
rs2967355
rs11117354
rs11085072
rs117512405
rs139795227
rs17445108
rs185174247
rs10905255
rs78491606
rs376641875
rs73730598
rs10774624
rs450962
rs12932179
rs1023767
rs2282764
rs9600019
rs9878436
rs10773176
rs10112752
rs429358
rs150150565
rs28577594
rs6054257
rs11212631
rs1957937
rs11579626
rs7221585
rs117034449
rs11584821
rs10845387
rs12369950
All − MR Egger
All − Inverse variance weighted
−2
−1
0
1
2
MR effect size for
'telomere length || id:ieu−b−4879' on 'IDP_dMRI_TBSS_OD_Uncinate_fasciculus_R
